# Molecular mechanisms and translational prospects in osteoporosis

**DOI:** 10.3389/fendo.2026.1907271

**Published:** 2026-07-22

**Authors:** Ping Xie, Jie Cai, Zhengjie Yu, Junjie Zhou, Jianping Wang

**Affiliations:** State Key Laboratory of New Targets Discovery and Drug Development for Major Diseases, Gannan Innovation and Translational Medicine Research Institute, Gannan Medical University, Ganzhou, China

**Keywords:** bone remodeling, molecular mechanisms, osteoporosis, research progress, therapeutic targets

## Abstract

Osteoporosis is the most prevalent chronic metabolic bone disease, affecting approximately 200 million individuals worldwide. With the rapid acceleration of global aging, osteoporosis has emerged as a major global health challenge, significantly increasing the occurrence rate of fractures and the associated medical burden. In recent years, considerable progress has been made in understanding the molecular mechanisms underlying osteoporosis, including bone remodeling, osteoblast-osteoclast communication, and signaling pathways such as RANKL/RANK/OPG and Wnt/β-catenin. These mechanistic studies have been translated into clinical applications. Emerging therapies, including targeted biologics and precision medicine approaches, offer novel strategies and methods for future prevention and treatment. This review summarizes recent advances in research, with a particular focus on therapeutic agents and mechanistic studies of osteoporosis. Following the mainline of “mechanism–target–translation,” this review proposes new research perspectives and approaches, and provides an outlook on future research directions for the disease, aiming to promote the development of more effective diagnostic and therapeutic strategies.

## 1 Introduction

Osteoporosis (OP) is a systemic skeletal disease resulting from an imbalance between bone resorption and bone formation (1). Globally, approximately 6.3% of men and 21.2% of women over the age of 50 are affected (2), and the number of cases is projected to double by 2040. Among patients who sustain a hip fragility fracture, 25% die within one year (3, 4). With the aging population, the annual medical costs associated with fractures are projected to increase by 121%, from $29.9 billion in 2020 to $65.9 billion in 2040, imposing a substantial socioeconomic burden (5, 6). Current clinical management faces challenges such as insufficient durability of therapeutic efficacy, limited improvement in bone quality, and a lack of personalized treatment. Long-term use of antiresorptive drugs carries the risk of atypical fractures, while bone-forming agents are constrained by treatment duration and cost. More importantly, osteoporosis exhibits profound heterogeneity, regulated by multiple hierarchical factors including genetics, endocrine signaling, mechanical loading, immunity, and aging. The traditional “bone-centric” perspective and single-target strategies are increasingly inadequate for precision intervention. In recent years, research in bone biology has advanced rapidly. The understanding of the regulation of bone remodeling has expanded from classical pathways, such as RANKL/RANK/OPG and Wnt/β-catenin, to novel dimensions including mechanosensing (e.g., Piezo1), epigenetic programming, organelle crosstalk, cellular senescence, energy metabolism reprogramming, and inter-organ communication (the “bone axis”) with the brain, gut, and liver. This progress has led to the emergence of innovative therapeutic targets, such as mechanoreceptors, RNA methylation modifications, and the clearance of senescent bone cells.

However, existing reviews predominantly focus on a single signaling pathway or a specific mechanism, lacking a systematic discussion of the cross-regulation and hierarchical integration across different mechanistic dimensions. Although significant breakthroughs have been achieved in mechanistic studies of osteoporosis, these advances have yet to be synthesized into a unified conceptual framework to guide target discovery and translational decision-making. To address this gap, the present review proposes a three-dimensional integrative perspective encompassing “mechanics–metabolism–epigenetics,” systematically delineating three emerging regulatory dimensions in bone remodeling (1): how mechanosensing pathways (Piezo1/2-YAP/TAZ) transduce physical signals into osteometabolic instructions (2); how energy metabolic reprogramming (glycolysis–oxidative phosphorylation switch, PRMT6 metabolic switch) determines the anabolic and catabolic capacities of bone cells; and (3) how epigenetic modifications (DNA methylation, histone modifications, m6A RNA methylation) regulate bone-related gene expression at the transcriptional and post-transcriptional levels. More importantly, this review, for the first time, systematically aligns these mechanisms with clinical-translational pathways by classifying targets according to their preclinical/clinical maturity and proposes an integrated framework encompassing “mechanism elucidation—target validation—translational assessment.” Through this framework, we aim to provide an actionable theoretical basis for the precision subtyping of osteoporosis and the development of next-generation therapeutic.

Three distinctive contributions distinguish this review from existing literature (1): We propose, for the first time, a “mechano–metabolic–epigenetic” tri-dimensional integrative framework that systematically examines the crosstalk among three emerging regulatory dimensions, rather than describing isolated pathways in a siloed manner (2). We classify the included studies by level of evidence, explicitly distinguishing between *in vitro*, animal, and human data, and clearly indicating the translational maturity of each mechanism discussed (3). We provide a map of therapeutic targets according to their preclinical or clinical maturity, systematically categorizing targets—including RANKL, Wnt, Piezo1, and m6A modifiers—by their developmental stage, thereby offering a clear priority framework for future drug development.

### 1.1 Methods

This is a narrative review based on a structured literature search. The MEDLINE database (via PubMed) was systematically searched for studies investigating the molecular mechanisms of osteoporosis. The search period encompassed publications from the database’s inception through 2026. The final search was performed on June 26, 2026. The complete database-specific search strategies are provided in **Supplementary Table 1**.

The search strategy incorporated controlled vocabulary, where applicable, and free-text terms relevant to osteoporosis, bone remodeling, molecular targeted therapy, the RANKL/RANK/OPG signaling pathway, the Wnt signaling pathway, the mechanosensing signaling pathway, DNA methylation, histone modifications, and RNA methylation. Medical Subject Headings (MeSH) were used for the PubMed search.

Two reviewers independently performed the literature search and screened titles, abstracts, and full texts for relevance. Included studies comprised original clinical research, cohort studies, case series, case reports, clinical trials, reviews, and mechanistic studies with information pertinent to osteoporosis mechanisms, bone remodeling, signal transduction, epigenetic regulation, or therapeutic targets. Studies were excluded if they were unrelated to osteoporosis, did not address the molecular mechanisms or signaling pathways of interest, were not published in English or Chinese, or were beyond the scope of this review. Discrepancies between the two reviewers were resolved through discussion; if consensus could not be reached, a third reviewer was consulted for final adjudication.

## 2 Bone remodeling and osteoporosis

Bone remodeling is a lifelong dynamic process that depends on the balance between osteoblasts (bone formation) and osteoclasts (bone resorption) to maintain bone metabolic homeostasis (7). With advancing age, bone resorption increases while bone formation becomes insufficient, leading to an imbalance in remodeling and consequently resulting in osteoporosis (8). Bone remodeling consists of three stages: osteoclast-mediated resorption, the reversal phase (recruitment of osteoblasts), and osteoblast-mediated formation (9).

Risk factors for osteoporosis include advanced age, menopause, a family history of osteoporosis, smoking, inadequate calcium intake, excessive alcohol consumption, insufficient physical activity, and long-term use of glucocorticoids (10, 11). Based on etiology, osteoporosis is classified into two major categories: primary and secondary (12, 13). Primary osteoporosis is associated with aging (including menopause-related bone loss in women) and is characterized by decreased bone mineral density and bone quality, reduced bone strength, and an increased risk of fracture (14, 15). It is further subdivided into three types (16). The first is postmenopausal osteoporosis (Type I), which is caused by estrogen deficiency and predominantly affects trabecular bone. The second is senile osteoporosis (Type II), which primarily occurs in individuals aged 75 years and older and results from age-related degeneration of both trabecular and cortical bone (17). The third is idiopathic osteoporosis, which affects younger adults and is highly heterogeneous; it is caused by decreased osteoblast activity and insufficient bone formation during the growth period, with sex hormone deficiency and cessation of ovarian function at menopause being major contributing factors (18). Currently, primary osteoporosis is the most prevalent form of the disease. Secondary osteoporosis is induced by underlying medical conditions or specific medications (e.g., glucocorticoids, which are the most common cause). It is characterized by bone loss, disruption of bone microarchitecture, increased bone fragility, and a heightened susceptibility to fracture (19–21). Underlying etiologies may include lifestyle modifications, genetic disorders, gastrointestinal diseases, thyroid dysfunction, hematological disorders, endocrine diseases, hypogonadism, rheumatic and autoimmune diseases, neuromuscular disorders, chronic disorders (affecting nutrient absorption or hormonal levels), and certain medications (5).

## 3 Research progress on the mechanisms of bone remodeling imbalance

### 3.1 RANKL/RANK/OPG axis

#### 3.1.1 Core signaling axis: RANKL/RANK/OPG and osteoclast regulation

The receptor activator of nuclear factor-κB ligand (RANKL)/Receptor Activator of Nuclear Factor-κB(RANK)/osteoprotegerin (OPG) signaling axis is established as the principal regulatory system governing osteoclast differentiation and bone resorption. Udagawa et al. provided a comprehensive framework demonstrating how this axis is modulated by auxiliary molecules—including OPG, sclerostin, leukemia inhibitory factor, and sialic acid-binding immunoglobulin-like lectin 15 (Siglec-15)—to maintain skeletal homeostasis (22). Two distinct preclinical strategies have been pursued to disrupt RANKL-RANK signaling, though with markedly different mechanisms and translational prospects. The first employs pharmacological inhibition of the RANKL-RANK interface: the pan-HDAC inhibitor vorinostat (SAHA) directly binds RANKL, blocking RANK interaction and suppressing downstream nuclear factor kappa-B (NF-κB)/mitogen-activated protein kinase (MAPK) signaling and nuclear factor of activated T cells c1 (NFATc1) activity, thereby inhibiting osteoclast differentiation *in vitro* and preventing bone loss in ovariectomized (OVX) mice (23). The second modulates the endogenous RANKL/OPG balance: β-sitosterol downregulates RANKL while upregulating OPG and promoting Runx2, leading to dose-dependent bone mass improvements in a glucocorticoid-induced osteoporosis rat model (24).

When evaluated collectively, these preclinical findings validate the RANKL-RANK interaction as a therapeutically tractable target. However, the evidence quality is uniformly preclinical. Both SAHA and BSS data derive exclusively from rodent or *in vitro* models, with no human data available. SAHA’s broad-spectrum HDAC activity raises selectivity and systemic toxicity concerns; BSS faces additional challenges in oral bioavailability, metabolite characterization, and the absence of human pharmacokinetic data. Moreover, OPG’s *in vivo* dynamics as a decoy receptor may vary across skeletal sites and pathological states (22).

The translational relevance of this axis is clinically validated by Denosumab, a fully human RANKL-neutralizing antibody approved by the Food and Drug Administration (FDA) in 2010. Denosumab reduces vertebral, non-vertebral, and hip fracture risk over 10 years of continuous use. However, its limitations—infection risk, hypocalcemia, rebound bone loss upon discontinuation, and the need for twice-yearly injections—underscore that even successful RANKL-targeting therapies require further refinement in specificity, delivery, and combination strategies to optimize long-term outcomes.

#### 3.1.2 Inhibitors and compounds targeting the RANKL-RANK interaction

Efforts to therapeutically target the RANKL-RANK interaction have diverged into two principal mechanistic strategies: direct blockade of the RANKL-RANK interface itself, and indirect suppression of osteoclastogenesis via multi-target natural products that modulate downstream signaling cascades. The evidence base, translational maturity, and specific limitations differ substantially between these two approaches.

Two natural compounds have been characterized as direct RANKL-binding agents, though their mechanisms of interaction are distinct. Chrysoeriol acts as a competitive inhibitor that engages the Lys-181 residue of RANKL, thereby preventing RANKL-RANK complex formation. This compound simultaneously suppresses NOX1-mediated reactive oxygen species (ROS) generation and modulates the nuclear factor erythroid 2-related factor 2 (NRF2)/Kelch-like ECH-associated protein 1 (KEAP1) antioxidant axis, leading to effective inhibition of osteoclast differentiation and bone resorption in both *in vitro* assays and OVX mouse models (25). Cichoric acid adopts a different binding mode, associating with RANKL at multiple sites rather than a single epitope; this engagement blocks downstream NF-κB and MAPK signaling, suppresses NFATc1 activation, and consequently inhibits osteoclastogenesis without detectable cytotoxicity *in vitro* (26). Both compounds directly target RANKL itself rather than downstream mediators—a feature that may theoretically confer higher pathway specificity—, but the precise binding epitopes, affinities, and structure-activity relationships of chicoric acid remain to be fully elucidated. A second category of natural products inhibits osteoclastogenesis through broader effects on autophagic, oxidative stress, and transcriptional pathways, rather than direct RANKL engagement. Curcumin suppresses RANKL-c-Jun N-terminal kinase (JNK)-BCL2-Beclin1 signaling, thereby inhibiting autophagy in osteoclast precursors and attenuating osteoclast differentiation in murine models (27). Piperlongumine blocks the p38/JNK-c-Fos/NFATc1 signaling cascade, suppressing osteoclastogenesis and preventing bone loss in OVX mice (28). Kurarinone targets NF-κB and MAPK pathways through extracellular signal-regulated kinase (ERK)/JNK phosphorylation inhibition (29). Agrimophol acts via the Blimp1-Bcl6 axis to suppress NFATc1 expression, reducing osteoclast differentiation in an inflammatory bone erosion model (30). When evaluated collectively, these findings indicate that both direct RANKL inhibitors and multi-target natural products can suppress osteoclastogenesis in preclinical systems, acting at different nodes of the RANKL signaling network. However, the quality and translational relevance of this evidence base are substantially limited. All data described above are derived exclusively from *in vitro* osteoclast differentiation assays or rodent OVX models; no human clinical data exist for any of these compounds. Direct RANKL binders such as chrysoeriol and chicoric acid have only been characterized at the molecular binding level, without pharmacokinetic profiling or *in vivo* efficacy data beyond single rodent studies. Multi-target natural products face additional challenges: their oral bioavailability is likely poor (though not formally assessed in most cases), their active metabolites and tissue distribution are unknown, and their target specificity—given the multiplicity of reported effects—remains uncertain. Moreover, the concentration ranges effective *in vitro* (often in the micromolar range) may not be achievable in human plasma without toxicity, a critical translational gap that has not been addressed in any of these studies.

The translational value of these compounds should therefore be viewed through an appropriate lens: they represent structurally interesting lead molecules for medicinal chemistry optimization, not clinical candidates in their current form. The favorable toxicity profiles reported in a few studies require cautious interpretation, given the short duration of exposure *in vitro* and the absence of any systematic toxicological evaluation in higher organisms. Future development pathways should prioritize (1): rigorous pharmacokinetic and pharmacodynamic characterization (2); medicinal chemistry optimization to improve potency, selectivity, and bioavailability (3); target engagement validation in relevant cell types using chemoproteomic approaches; and (4) systematic toxicological evaluation in long-term animal models. As outlined in Section 3.1.6, the broader clinical development landscape for RANKL-targeting agents—including biosimilars, peptide inhibitors, small molecules, and vaccines—provides a contextual roadmap, though the natural products discussed here remain at the earliest stages of this translational trajectory.

#### 3.1.3 Regulation of ROS, autophagy, and epigenetic signaling pathways

Several mechanistically distinct strategies converge on the suppression of RANKL-induced osteoclastogenesis through ROS modulation and epigenetic regulation. Two compounds directly target ROS pathways: CHE inhibits NOX1-mediated ROS generation while modulating the NRF2/KEAP1 antioxidant axis, and dictamnine acts through a dual mechanism involving both ROS clearance and NF-κB pathway inhibition (31). The cannabinoid receptor type 2 (CB2) antagonist AM630 offers a pharmacologically distinct approach, suppressing RANKL-induced osteoclast differentiation by upregulating Nrf2 and its downstream target heme oxygenase-1 (HO-1) (32). Beyond ROS-targeting mechanisms, an epigenetic dimension has emerged through the osteoblast-derived histone demethylase Jmjd3, which regulates osteoclastogenesis indirectly by modulating EphB4 and RANKL expression in osteoblasts, thereby mediating osteoblast-osteoclast communication (33).

Collectively, these findings indicate that ROS modulation and epigenetic regulation each suppress osteoclastogenesis via distinct pathways. However, the evidence remains exclusively preclinical—all findings derive from *in vitro* osteoclast differentiation models or OVX mice, with no human data available. The cell type-specific functions of Jmjd3 in bone tissue also require further confirmation through conditional knockout studies.

#### 3.1.4 Novel targets and mechanisms of calcium signaling, mitochondria, and mechanosensing

Three distinct mechanistically informed strategies have emerged for targeting osteoclastogenesis through calcium and mitochondrial signaling. The first operates through mechanical coupling: the Piezo1-Ca²^+^-YAP axis in osteoblasts senses mechanical loading and suppresses osteoclastogenesis by downregulating RANKL and upregulating OPG expression (7). The second involves pharmacological modulation of mitochondrial calcium flux: ruthenium red, and mitochondrial calcium uniporter (MCU) inhibitor, simultaneously suppresses osteoclastogenesis and promotes osteogenesis by inhibiting ROS and the p38 MAPK/NFATc1 axis (34). The third, surprisingly, identifies a negative regulatory role for calcium signaling: transient receptor potential vanilloid 6 (TRPV6) deletion promotes osteoclast differentiation and bone resorption through insulin-like growth factor (IGF)- phosphoinositide 3-kinase (PI3K)- protein kinase B (AKT) pathway hyperactivation, resulting in an osteoporotic phenotype (35).

Collectively, these findings establish that calcium signaling and mitochondrial function operate at multiple levels to modulate osteoclast biology. However, the mechanistic and translational gaps are substantial: the molecular crosstalk between Piezo1-mediated mechanoregulation and TRPV6-mediated calcium signaling remains unexplored; RR’s target specificity as an MCU inhibitor requires systematic evaluation; and the clinical relevance of TRPV6 variants in human osteoporosis has not been investigated.

#### 3.1.5 Multi-target natural products and clinical translation potential

Three additional agents with anti-osteoclastogenic activity have been characterized in preclinical models, each acting through distinct mechanisms. Zanthoxylum bungeanum seed oil suppresses RANKL-induced osteoclastogenesis by inhibiting the ERK/c-JUN/NFATc1 pathway and inducing cell cycle arrest in RAW264.7 cells (36). Chrysosplenetin, a flavonoid, reduces osteoclast differentiation and bone resorption by targeting RANKL-induced NF-κB signaling and NFATc1 activation (37). The tetravalent peptide WHD-tet employs a distinct mechanism, concurrently blocking RANK–TNF receptor-associated factor 6 (TRAF6) multivalent binding and restoring bone mineral density (BMD) by 28% in OVX rats without immunosuppression (38).

All three agents demonstrate anti-osteoclastogenic activity in preclinical models, but the evidence is uniformly preclinical—derived from cell lines or short-term OVX models, with no human data available. Critical gaps include unestablished extraction and quality control protocols, unknown oral bioavailability and tissue distribution, and unverified long-term safety. These compounds should therefore be viewed as structural leads for optimization rather than clinical candidates.

#### 3.1.6 Clinical translation landscape and future development directions

The RANKL/RANK/OPG axis supports a diversified clinical pipeline beyond the approved antibody Denosumab. Biosimilars—including KN012, SB16, RGB-14-P, and SPD8—have completed or are undergoing Phase III trials, showing comparable efficacy and safety to the reference product, though long-term immunogenicity data are still accumulating. Peptide inhibitors derived from RANKL fragments have demonstrated dual preclinical activities—inhibiting osteoclastogenesis while reducing sclerostin expression—but oral bioavailability and *in vivo* stability remain major hurdles. Small-molecule inhibitors (e.g., IMB-R38) and derivatives have shown nanomolar activity *in vitro*, yet protein-protein interaction interfaces lack well-defined binding pockets, hindering translation. Vaccine strategies targeting RANKL have shown bone loss prevention in transgenic mice, though human safety and efficacy await validation.

**Figure 1:**
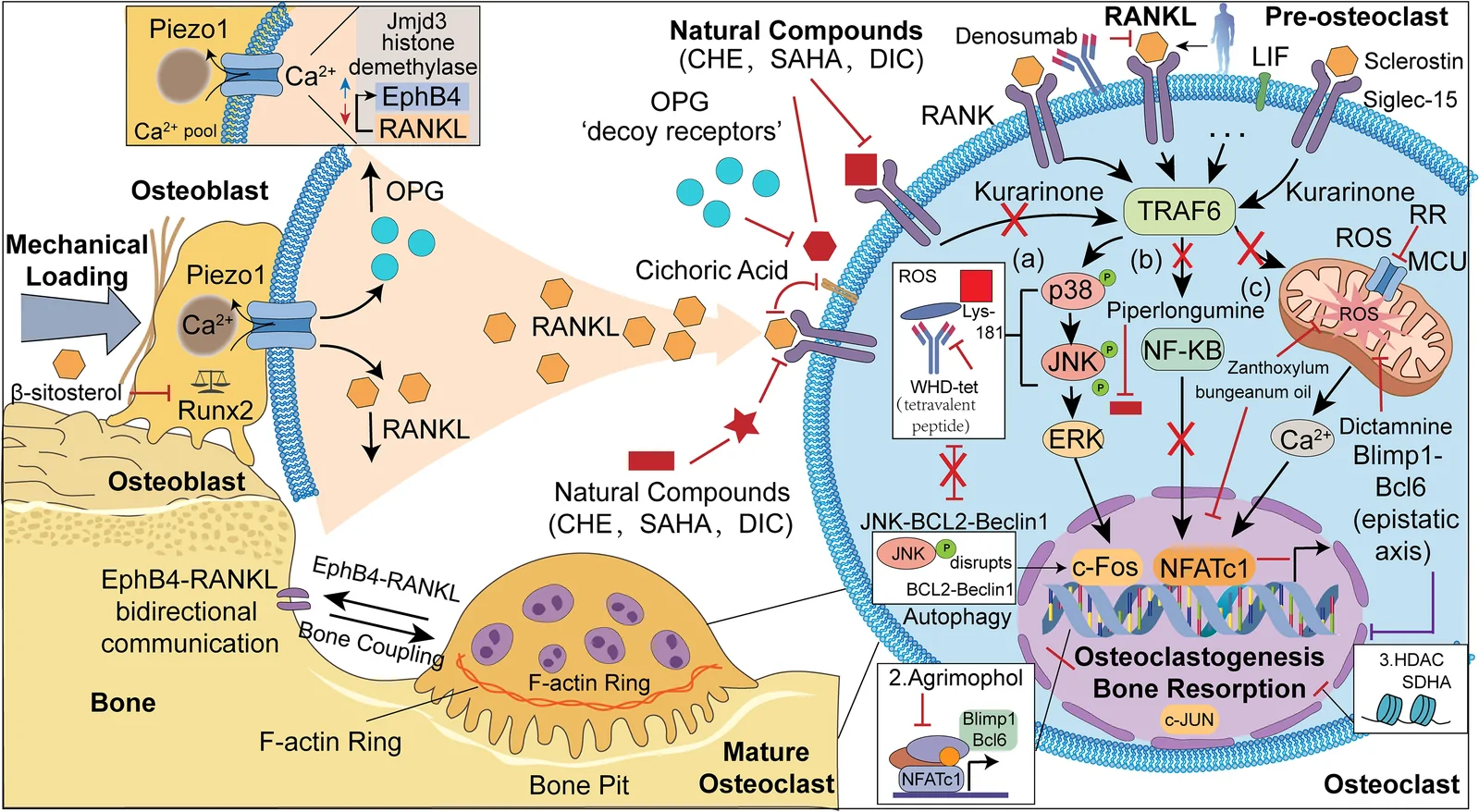
The RANKL/RANK/OPG signaling axis regulates osteoclastogenesis and the intervention mechanisms of natural products. This figure illustrates the RANKL/RANK/OPG axis in osteoclast differentiation (1): Mechanical stress modulates RANKL/OPG via Piezo1 (2); RANKL-RANK activates NF-κB/MAPK to drive c-Fos/NFATc1 and Blimp1-Bcl6 (3); Natural compounds target key nodes to block osteoclastogenesis; (4) Mature osteoclasts resorb bone via F-actin ring and signal via EphB4-RANKL.

Each modality faces distinct barriers: biosimilars require long-term immunogenicity surveillance; peptides and small molecules must overcome bioavailability and targeting challenges; vaccines need human proof-of-concept. Future directions should prioritize next-generation RANKL biologics with extended dosing and improved safety, synergistic strategies combining RANKL/RANK/OPG with Wnt or Piezo1 pathways, and investigation of gut microbiota and neuroendocrine regulation of the RANKL/OPG axis for non-skeletal-targeted interventions (**Figure 1**).

### 3.2 Wnt/β-catenin signaling

#### 3.2.1 Core signaling axis

Canonical Wnt/β-catenin signaling is initiated by Wnt binding to low-density lipoprotein receptor-related protein 5/6 (LRP5/6) and Frizzled, which inhibits glycogen synthase kinase-3 beta (GSK3β)-mediated β-catenin degradation, promotes β-catenin nuclear translocation, and activates T-cell factor (TCF)/lymphoid enhancer-binding factor (LEF)-driven osteogenic gene expression. This core pathway is subject to multi-layered regulation by both positive and negative modulators.

Positive modulators that enhance Wnt signaling include ZBP1, which promotes β-catenin nuclear translocation through a positive feedback loop (39); BRD4, an epigenetic reader that upregulates Wnt3a/β-catenin expression (40); CDK14, which activates Wnt via LRP6/GSK3β phosphorylation (41); and GNAS, which promotes osteogenesis through Wnt activation (42). The Wnt3a-LRP6-mTORC1-β-catenin cascade has also been elucidated as a key signaling module in osteoblast differentiation (43). Negative modulators include Foxf1, which is elevated in OVX mice and suppresses Wnt activity (44), and LECT2, whose knockdown activates Wnt to promote mesenchymal stem cell (MSC) osteogenesis (45). A critical antagonistic relationship also exists between canonical and non-canonical Wnt signaling: β-catenin nuclear translocation initiates osteogenesis, whereas activation of the Wnt5a-CaMKII axis drives adipogenesis (46).Collectively, these findings establish that Wnt/β-catenin signaling controls MSC osteogenic-adipogenic fate determination through multiple positive and negative regulatory nodes. However, the translational significance of these modulators remains uncertain: most findings are derived from *in vitro* or single-animal models, their consistency across skeletal sites is unknown, and the long-term off-target effects of GSK3β inhibition—a downstream consequence of pathway activation—warrant careful evaluation.

#### 3.2.2 Integrated regulation of Wnt signaling: pharmacological modulators and cellular networks

Rather than functioning as an isolated linear cascade, Wnt/β-catenin signaling is modulated by a highly integrated network involving pharmacological activation, cellular stress responses, and post-translational modifications.

Pharmacological Targeting and Natural Modulators: Small molecule interventions primarily target the destruction complex or upstream stabilizing factors. For instance, the GSK3β inhibitor CHIR99021 stabilizes β-catenin while concurrently inducing autophagy, illustrating a permissive crosstalk between autophagic clearance and Wnt activation (47). Similarly, the HIF stabilizer roxadustat activates Wnt secondary to hypoxia-inducible factor 1-alpha (HIF-1α) stabilization (48). A diverse array of natural products converges on this pathway; compounds such as icariin (49), Cistanche extracts (50), and bergamottin (51) activate Wnt signaling via direct GSK3β suppression or epigenetic modulation. Additional plant-derived agents, including Total Flavonoids of Rhizoma Drynariae (52), barbaloin (53), aucubin (54), and daphnetin (55), systematically upregulate osteogenic targets or suppress antagonistic signals like Wnt5a. Multi-component formulations like Qi-Gui-Jian-Gu decoction (56) and tuna bone powder (57) further demonstrate network-level pathway activation. However, most of these natural products remain limited by poor pharmacokinetic characterization and a lack of human data.

Crosstalk with Autophagy, Oxidative Stress, and Epigenetics: The Wnt network is intricately tied to cellular stress and epigenetic regulation. Pathological pathway activation can occur via fluoride exposure, which triggers autophagy and Wnt signaling through HIF-1α, leading to abnormal osteosclerosis rather than physiological bone formation (58). Furthermore, oxidative stress engages a regulatory feedback loop with Wnt signaling through epigenetic modifiers like METTL3, balancing osteoblast survival and apoptosis (59). Epigenetic control is also mediated by non-coding RNAs; MSC-derived exosomes deliver miR-27a to relieve Wnt blockade (60), while miR-486-3p directly targets antagonists to activate the pathway (61). Additionally, traditional formulations like Liuwei Dihuang Pill have been shown to modulate this epigenetic-Wnt axis to improve bone properties (62).

Post-translational Fine-Tuning and Novel Regulators: Beyond pharmacological and epigenetic modulation, the pathway is fine-tuned by an expanding network of post-translational modifiers. Positive regulators such as Rspo1 (63), ADM2 (64), MTSS1 (65), and ANKRD1 (66) potentiate Wnt signaling by stabilizing β-catenin or suppressing antagonistic pathways. Conversely, negative regulators like FKBP5 (67) and FOXO4 (68) restrict pathway activity by promoting β-catenin ubiquitination or disrupting the osteogenic-adipogenic balance.

Translational Considerations: While these integrated networks offer multiple druggable nodes, sustained Wnt activation poses severe extra-skeletal safety risks. This is exemplified by romosozumab, the FDA-approved Wnt pathway-targeting therapy. Although it demonstrates robust fracture risk reduction, its boxed warning for cardiovascular events underscores the critical need for tissue-specific Wnt modulators and careful patient stratification before any novel Wnt-activating agents can transition to clinical use.

#### 3.2.3 Exosomes, stem cells, and bone tissue engineering

Exosome-based strategies offer a cell-free approach to Wnt pathway activation. Lithium promotes the secretion of Wnt10a-enriched exosomes via the MARK2-Rab11a axis, activating Wnt signaling in recipient cells (69). The role of MSC-derived exosomes in delivering miR-27a to suppress DKK2 has been discussed in the context of epigenetic regulation (see Section 3.2.3) (60).

**Figure 2:**
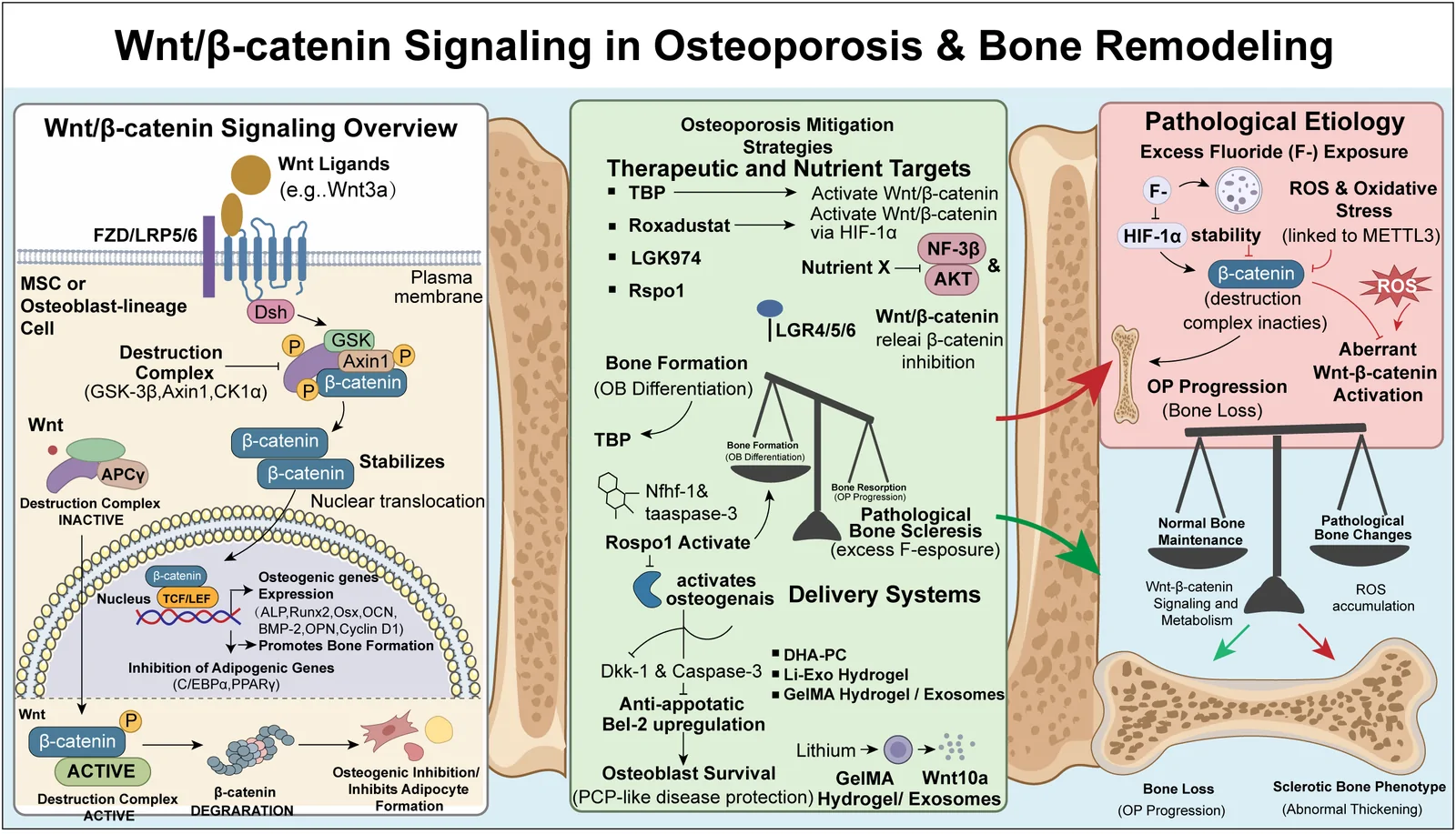
Regulation of bone metabolism by the Wnt/β-catenin signaling pathway and its interventional mechanisms. This figure outlines Wnt/β-catenin in bone (1): Wnt inhibits GSK-3β, enabling β-catenin nuclear translocation to drive Runx2/OCN for osteogenesis; (2) F^-^disrupts this via ROS/HIF-1α, causing imbalance; (3) Activators (TBP, roxadustat) and inhibitors (LGK974) modulate osteoblast-osteoclast crosstalk, alongside delivery systems like DHA-PC and Li-Exo.

These strategies offer novel approaches for targeted Wnt pathway activation, but significant translational barriers remain: standardized exosome production, quality control, dosing, and long-term safety are unresolved; the biocompatibility and degradation behavior of 3D hydrogel materials require further optimization; and none have progressed to clinical evaluation (**Figure 2**).

### 3.3 Energy Metabolism reprogramming

Energy metabolism reprogramming in bone cells switches pathways (glycolysis, oxidative phosphorylation, fatty/amino acid metabolism) to maintain remodeling; dysregulation leads to bone loss.

#### 3.3.1 Key regulatory mechanisms of osteoblast metabolic reprogramming

Osteoblasts rely primarily on aerobic glycolysis to meet the high energy demands of bone matrix synthesis. Mechanistically, this metabolic preference is supported by at least four distinct regulatory nodes. At the mitochondrial level, malic enzyme ME2 couples mitochondria to aerobic glycolysis via the malate-aspartate shuttle, sustaining osteoblast proliferation and differentiation (70). At the signaling integration level, bone morphogenetic protein (BMP) signaling coordinates glucose, fatty acid, and amino acid metabolism through the mTOR-HIF-Wnt-autophagy network to maintain bone homeostasis (71). At the post-translational modification level, Wnt signaling elevates O-GlcNAcylation via the Ca²^+^-PKA-Gfat1 axis, stabilizing PDK1 to enhance glycolysis and bone formation; loss of this modification suppresses Wnt-induced glycolysis and delays fracture healing (72). At the exogenous regulatory level, Toxoplasma gondii excreted/secreted proteins (TgESPs) promote bone marrow mesenchymal stem cell (BMSC) aerobic glycolysis and osteogenic gene expression via Wnt/β-catenin activation (73).

Collectively, these findings establish aerobic glycolysis as central to osteoblast energy supply, with multiple regulatory layers converging on this metabolic program. However, key translational gaps persist: the conservation of ME2- and O-GlcNAcylation-mediated mechanisms in human osteoblasts remains unconfirmed; the immunogenicity of TgESPs precludes therapeutic application. Notably, diabetic metabolic disorders impair osteoblast glycolysis and bone turnover, and type 1 diabetes-induced bone loss can be rescued by glycolysis activation, positioning osteoblastmetabolic reprogramming as both a fundamental formation mechanism and a pathological bridge linking systemic metabolic diseases to skeletal complications.

#### 3.3.2 Key regulatory mechanisms of osteoclast metabolic reprogramming

Osteoclast differentiation and bone resorption depend on a distinct metabolic program. At the glucose metabolism level, Glut1-mediated glucose uptake drives osteoclastogenesis through both aerobic glycolysis and oxidative phosphorylation (74). At the epigenetic level, protein arginine methyltransferase 6 (PRMT6) suppresses fatty acid oxidation while promoting glycolysis to drive osteoclast differentiation; its deletion reverses this metabolic switch and ameliorates OVX osteoporosis (75). At the functional execution level, osteoclast bone resorption specifically requires aerobic glycolysis and lactate production, as glycolysis inhibition blocks resorption without affecting cell viability (76). Additionally, osteoclasts generate ATP through both glycolysis and oxidative phosphorylation, regulated by epigenetic and systemic metabolic cues, with compensatory mechanisms proposed to reset energy balance (77).

Collectively, these findings establish glycolysis as a critical and therapeutically targetable energy source for osteoclast bone resorption. However, this strategy faces dual challenges: systemic glycolysis inhibition risks immunosuppression and gastrointestinal toxicity due to the ubiquitous nature of glycolysis; the PRMT6-targeted approach and compensatory mechanisms remain at the proof-of-concept stage, with drug development and delivery efficiency unresolved.

#### 3.3.3 Clinical translation landscape and future directions

Despite the growing mechanistic understanding of metabolic reprogramming in bone cells, clinical translation remains in its infancy. To date, no drugs targeting osteoblast or osteoclast metabolism have been approved for osteoporosis treatment. The most advanced candidates are glycolysis inhibitors (e.g., 2-deoxyglucose derivatives) and PRMT6 inhibitors, both of which have shown efficacy in preclinical OVX models but have not yet entered clinical trials (75, 76). The primary translational barriers include (1): target specificity—glycolysis is a ubiquitous pathway shared by immune cells and rapidly proliferating tissues, raising concerns about off-target immunosuppression and gastrointestinal toxicity (2); drug delivery—achieving bone-selective metabolic modulation remains a formidable challenge; and (3) biomarker development—no validated metabolic biomarkers exist to monitor target engagement or predict therapeutic response.

Future directions should prioritize: (1) developing bone-targeted glycolysis inhibitors or PRMT6-selective modulators with improved safety profiles; (2) identifying predictive metabolic biomarkers (e.g., circulating lactate, glycolytic enzyme levels) for patient stratification; and (3) exploring combination strategies that integrate metabolic modulation with established therapies (e.g., RANKL inhibitors or Wnt activators) to achieve synergistic effects. The convergence of metabolic reprogramming with epigenetic regulation (e.g., PRMT6-mediated histone methylation) also offers opportunities for dual-targeting approaches (75).

### 3.4 The role of mechanosensory signal pathways in bone remodeling

Mechanochemical coupling converts mechanical stimuli into biochemical signals to regulate bone formation and resorption, a key homeostatic mechanism.

#### 3.4.1 Calcium signaling mediated by Piezo1/2 and downstream transcriptional regulation

Piezo1/2 are the core mechanosensors in bone cells, operating at multiple levels to transduce physical stimuli into osteogenic signals. At the channel function level, Piezo1/2 sense fluid shear stress and matrix stiffness to mediate Ca²^+^influx, activate calcineurin, and induce dephosphorylation and nuclear co-activation of NFATc1, YAP1, and β-catenin, driving osteogenic differentiation; channel deletion causes spontaneous fractures, impaired osteogenesis, and unloading resistance (78). At the synergistic activation level, fluid shear stress and hydrostatic pressure cooperatively promote BMSC osteogenesis via integrin-cytoskeleton and Piezo1-activated YAP1/NFAT2 nuclear translocation (79). At the molecular anchoring level, Piezo1 is tethered to the actin cytoskeleton via the cadherin-β-catenin complex, enabling force-gated channel activity (80). At the systematic level, calcium channels, including Piezo and voltage-sensitive channels, regulate osteogenesis through Yes-associated protein (YAP)/transcriptional coactivator with PDZ-binding motif (TAZ) and Wnt signaling (81).

Piezo1 also exhibits cell stage-specific functions: mature osteocyte-specific Piezo1 knockout reduces bone mass but preserves mechanical response, indicating developmental-stage decoupling (82). Pharmacologically, cyclic stretching (15%) upregulates osteogenic factors via Piezo1; the agonist Yoda1 enhances this effect, while the inhibitor GsMTx4 only partially blocks osteogenesis (83). Hyperbaric oxygen promotes bone regeneration by mimicking mechanical signals to activate Piezo1-YAP (84).

Collectively, these findings establish Piezo1/2-YAP/β-catenin as the core mechano-osteogenic coupling axis. However, translational barriers are substantial: available Piezo1/2 modulators lack subtype selectivity and bioavailability, with none approved for human use; optimal parameters for hyperbaric oxygen and mechanical stimulation remain undefined.

#### 3.4.2 Synergistic activation of the YAP/NFAT axis by multiple mechanical stimuli

Multiple mechanical stimuli converge on YAP/NFAT activation through distinct but interconnected pathways. Fluid shear stress and hydrostatic pressure synergistically activate YAP1/NFAT2 nuclear translocation via integrin-cytoskeleton and Piezo1 (79). Uniaxial cyclic stretch activates Wnt/β-catenin through YAP nuclear translocation in OPLL cells (85). In integrin-mediated mechanotransduction, fluid shear stress co-cultured with endothelial cells promotes BMSC osteogenesis through the integrin β1- focal adhesion kinase (FAK)-ERK1/2 axis (86). Piezo1 is mechanically coupled to the cytoskeleton via the cadherin-β-catenin complex (80).

These findings construct a complete signaling chain from mechanical stimulation to nuclear transcriptional activation. However, the crosstalk between integrin-FAK and Piezo1 pathways remains poorly understood; optimal mechanical parameters for different cell types have yet to be established, limiting rational mechano-therapeutic design.

#### 3.4.3 Integrin/adhesion complexes and cytoskeleton coupling

Macrophage Piezo1 mediates load-induced bone regeneration: mechanical loading promotes macrophage polarization toward the CD206^+^phenotype via Piezo1, enhancing angiogenesis-osteogenesis coupling and bone defect repair; myeloid-specific Piezo1 deletion abolishes this effect (87). This study expands mechanosensing to immune cells. However, the downstream signaling coupling macrophage Piezo1 to endothelial cells and osteoprogenitors, and the stability of this mechanism under inflammatory conditions, remain unclear.

#### 3.4.4 Immune cell-mediated mechanical regulation and angiogenesis-osteogenesis coupling

Mechanically stimulated BMSC-derived exosomes activate Wnt/β-catenin by upregulating LRP6/β-catenin/TCF7 and suppressing GSK-3β, reversing dexamethasone-suppressed osteogenesis (88). Bioinformatics analysis has identified 222 PIEZO1-regulated genes, including Lcn2 and Dkk3, enriched in Wnt/β-catenin and PI3K-Akt pathways (89). Exosome-based strategies offer a cell-free mechanotherapy approach but face challenges in standardized production and targeted delivery; bioinformatically predicted targets require functional validation, and the pathophysiological relevance of the PIEZO1 network in human bone needs confirmation.

#### 3.4.5 Exosomes and bioinformatics networks

The mechanosensing pathway has no approved therapeutics targeting Piezo channels or YAP/TAZ to date. Primary translational barriers include (1): lack of selective modulators—available Piezo1/2 agonists (e.g., Yoda1) and inhibitors (e.g., GsMTx4) lack subtype selectivity and have poor pharmacokinetic profiles (83) (2). delivery challenges—mechanical stimulation approaches (exercise, vibration, hyperbaric oxygen) are used clinically, but optimal parameters and patient-specific responses remain undefined (84); and (3) mechanism uncertainty—integration of Piezo1 with other mechanosensitive pathways requires further elucidation.

**Figure 3:**
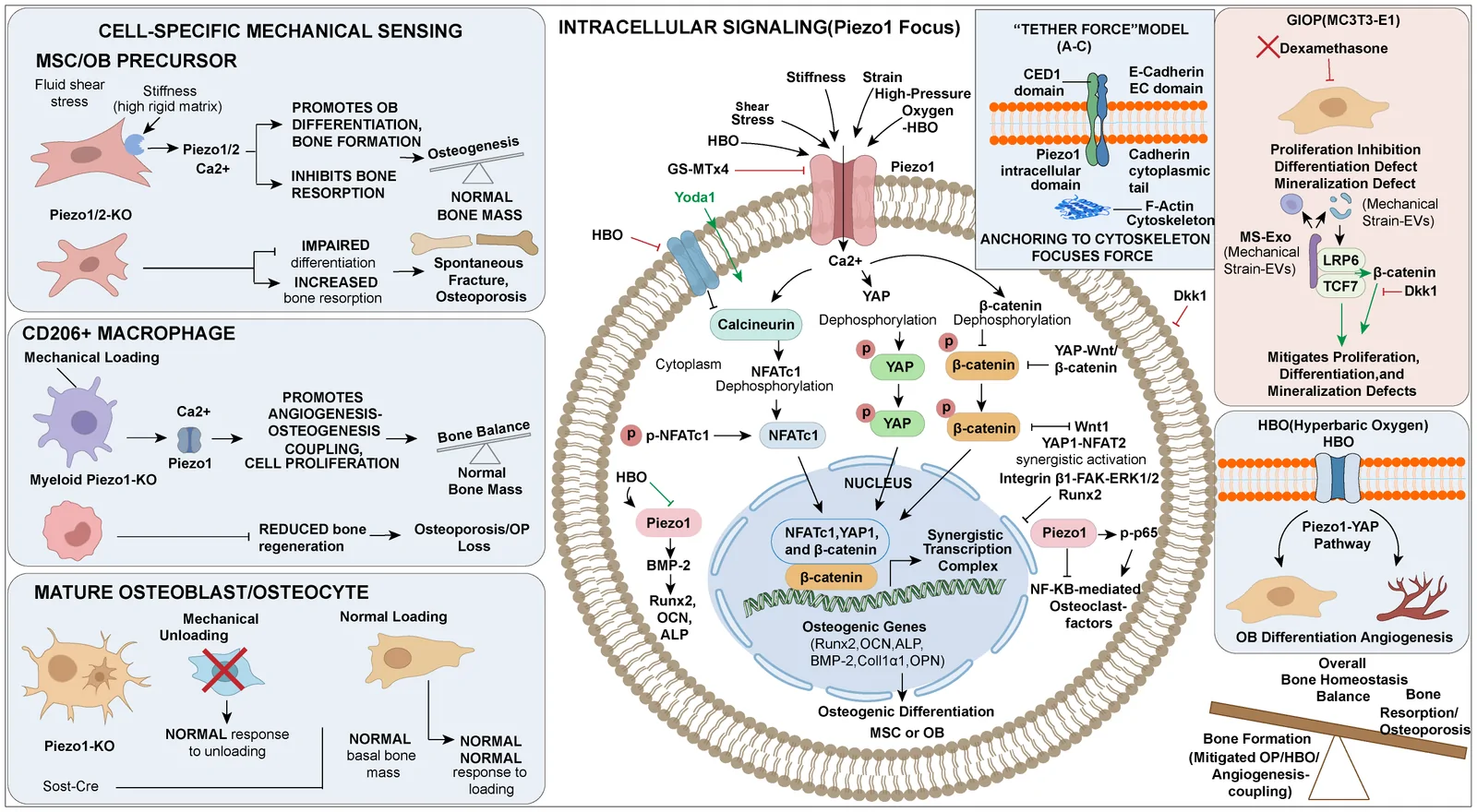
Piezo1/2-mediated mechanotransduction and regulation of bone homeostasis. This figure depicts Piezo1/2 in bone: (1) Fluid shear stress → Ca²^+^influx → activates YAP/NFATc1/β-catenin to drive osteogenesis; (2) Couples mechano-signals across MSCs, macrophages, and endothelium to balance bone formation/resorption; (3) Dexamethasone impairs mechanical-EV transfer, while hyperbaric oxygen rescues via Piezo1-YAP.

Future directions should focus on: (1) next-generation Piezo1/2 modulators with improved selectivity and bone-targeting; (2) rigorous trials to define optimal mechanical stimulation protocols for different populations; (3) Piezo1 agonists as adjuncts to pharmacological therapy; and (4) investigation of Piezo1 crosstalk with inflammatory and metabolic pathways to identify combinatorial targets (**Figure 3**).

## 4 Epigenetic regulation of bone

Epigenetic mechanisms in bone remodeling do not operate in isolated silos; rather, they form a highly integrated, multi-layered regulatory network. The orchestration of osteoblast and osteoclast differentiation relies on the dynamic crosstalk among DNA methylation, histone modifications, and RNA methylation. For instance, DNA methyltransferases (DNMTs) and histone deacetylases (HDACs) frequently cooperate within the same repressor complexes to coordinate chromatin condensation and silence key osteogenic genes. Simultaneously, RNA methylation (m6A) adds another layer of complexity by post-transcriptionally regulating the stability and translation of transcripts encoding these very epigenetic enzymes. Furthermore, active histone marks can dictate chromatin accessibility, thereby guiding DNA methylation patterns during MSC fate determination. Understanding this “epigenetic interactome” is clinically critical, as the pathological disruption of one layer in osteoporosis inevitably cascades into the others. The following sections delineate how these three primary epigenetic dimensions operate both individually and synergistically to govern skeletal homeostasis.

### 4.1 DNA methylation

DNA methylation is increasingly implicated in age-related bone diseases like osteoporosis. Physiologically, methylation represses transcription while demethylation promotes it; their imbalance disrupts bone immune homeostasis. Mechanisms involve: (1) regulating key bone metabolism genes/signaling, and (2) aberrant methylation in immune cells.

#### 4.1.1 DNMTs-mediated hypermethylation and osteogenic inhibition

DNA methyltransferases (DNMTs) suppress osteogenesis through at least three distinct but converging mechanisms. At the level of DNMT1-mediated direct transcriptional repression, DNMT1 is upregulated in bone tissues from senile osteoporosis patients and mice, where it binds to the RORA and Fgfr2 promoters and maintains their hypermethylation, thereby suppressing MSC osteogenic differentiation; DNMT1 knockdown reverses this repression (90). At the level of DNMT3b/3l pathology, upregulation of DNMT3b/3l combined with ten-eleven translocation 1 (TET1) downregulation in prematurely aged BMSCs causes genome-wide hypermethylation, particularly at Bmp2 and Fgfr2 promoters, suppressing Wnt activity and osteogenic capacity (91). Additionally, DNMTs also contribute to osteogenic suppression through glutathione peroxidase 4 (GPX4) silencing and ferroptosis, as detailed in Section 4.1.2 (92, 93).

Collectively, these findings establish that distinct DNMT family members—DNMT1, DNMT3a, and DNMT3b/3l—cooperatively inhibit osteogenesis through different target genes (RORA/Fgfr2, Gpx4, Bmp2). However, key questions remain: do these DNMTs exhibit synergistic or compensatory functions across different pathological contexts? Current evidence is largely derived from single-gene knockout or inhibitor studies, and no study has systematically compared their relative contributions.

#### 4.1.2 GPX4 hypermethylation mediated by DNMTs and ferroptosis of osteoblasts

A specific pathway linking DNA methylation to osteoblast cell death has been identified: DNMT1/3a (or DNMT1/3a/3b) upregulation in diabetic osteoporotic mice and OVX mice leads to Gpx4 promoter hypermethylation, suppressing GPX4 transcription and triggering osteoblast ferroptosis. Pharmacological intervention with Asperosaponin VI (AVI) or the DNMT inhibitor SGI-1027 reverses Gpx4 hypermethylation, restores GPX4 expression, and alleviates bone loss (92, 93).

This cascade, linking DNMTs-mediated GPX4 silencing to osteoblast ferroptosis and subsequent bone loss, illustrates the convergence of epigenetic regulation, oxidative stress, and programmed cell death in osteoporosis pathogenesis. However, the translational relevance of this pathway remains uncertain, as its operation in human osteoporotic bone has yet to be confirmed. Furthermore, the pharmacokinetic profiles and long-term safety of Asperosaponin VI and the DNMT inhibitor SGI-1027 require systematic evaluation before their clinical application can be considered.

#### 4.1.3 TET-mediated demethylation promotes osteogenic differentiation

TET-mediated DNA demethylation is a critical initiator of osteoblast differentiation. Pharmacological inhibition of TET with BC339 increases global 5mC and decreases 5hmC levels, leading to Sp7 promoter hypermethylation and transcriptional repression, which suppresses osteogenic marker gene expression and **ALP** activity; SP7 overexpression rescues this effect (94).

This loss-of-function study validates the necessity of TET-mediated demethylation for osteogenesis. However, the specificity of BC339 as a tool compound and its inhibitory profile across TET1/2/3 remain unclear. More importantly, the dynamic balance between TET and DNMT—how the “methylation-demethylation cycle” precisely controls osteogenic gene expression—represents a key unanswered question in epigenetic bone research.

#### 4.1.4 Key transcription factors and signaling pathways regulated by DNA methylation

At the antioxidant response level, aberrant DNA methylation reduces Nrf2 expression, correlating with OP pathogenesis; running exercise restores Nrf2 through epigenetic mechanisms and protects bone in OVX mice (95). At the osteogenic-adipogenic balance level, genome-wide methylation analysis has identified ZEB1 and ZEB2 as key methylation-regulated transcription factors controlling MSC fate, with ZEB1 positively correlated with obesity-related bone markers (96).

These findings extend DNA methylation regulation from gene promoters to transcription factor networks. However, the clinical relevance of Nrf2 promoter methylation in human OP requires validation; the downstream targets of ZEB1/2 in bone metabolism warrant further investigation.

From a clinical translation perspective, DNMT inhibitors (Decitabine, Azacitidine) are FDA-approved for oncology, but their application in osteoporosis remains unexplored. Key barriers include (1): target specificity—DNMTs and TETs act genome-wide, and bone-selective delivery is undeveloped; (2) safety—long-term epigenetic reprogramming carries risks of genomic instability; (3) biomarkers—no validated markers exist to monitor bone-specific target engagement; and (4) delivery—achieving sufficient bioavailability in bone tissue remains challenging. Future directions include bone-targeted DNMT inhibitors, epigenetic biomarkers for patient stratification, combination strategies with bone-active agents, and single-cell resolution studies to identify cell-type-specific vulnerabilities (**Table 1**).

**Table 1:** DNA methylation.

Category	Key regulatory	Main mechanisms and effects
DNMTs-mediated hypermethylation and inhibition of osteogenesis	DNMT1	In senile osteoporosis, DNMT1 hypermethylates RORA/Fgfr2 to suppress MSC osteogenesis (90).
	DNMT1/3a、DNMT1/3a/3b	In diabetic/OVX mice, Gpx4 hypermethylation suppresses GPX4 and triggers osteoblast ferroptosis (92, 93).
	DNMT3b/3l	In aged mouse BMSCs, Bmp2/Fgfr2 hypermethylation inhibits Wnt and osteogenesis (91).
DNMTs-mediated GPX4 hypermethylation and ferroptosis	DNMT1/3a and intervention	AVI or SGI-1027 reverses Gpx4hypermethylation, restoring GPX4 and reducing bone loss (92, 93).
TET-mediated demethylation promotes osteogenesis	TET activity	BC339 hypermethylates Sp7 via 5mC/5hmC shift; SP7 overexpression rescues (94).
	Nrf2	Methylation reduces Nrf2; RE restores it and protects OVX bone (95).
DNA Methylation Regulates Key Transcription Factors and Signaling Pathways	ZEB1/ZEB2	Methylation-regulated ZEB1/2 control MSC osteogenic-adipogenic balance; ZEB1 correlates with obesity-related bone metabolism (96).

### 4.2 Histone modifications

Histone modifications regulate chromatin structure and gene transcription through multiple chemical modifications, including methylation, acetylation, ubiquitination, and ADP-ribosylation, playing critical roles in osteoblast and osteoclast differentiation. Their dysregulation contributes to osteoporosis pathogenesis through three interconnected mechanisms: (1) the histone demethylase KDM7A bidirectionally regulates osteoblast and osteoclast differentiation by removing repressive histone marks from Fap and Rankl promoters; (2) histone acetylation promotes osteogenesis via p300-mediated Runx2 acetylation, whereas HDAC11-mediated deacetylation suppresses BMSC osteogenesis through transgenerational programming; and (3) the coordinated switch between acetylation and arginine methylation creates a transcriptionally active chromatin environment in vitamin D signaling. These three layers represent the regulatory logic of histone modifications in “gene activation–gene silencing,” “transcriptional promotion–transcriptional repression,” and “coordinated modification switching,” collectively constituting a multi-level regulatory network governing bone metabolism.

#### 4.2.1 Histone methylation modification

The histone demethylase KDM7A exemplifies a unique bidirectional regulatory mechanism: it removes repressive H3K9me2 and H3K27me2 marks from Fap and Rankl promoters, thereby upregulating FAP and RANKL expression. This dual action simultaneously suppresses osteogenic differentiation and bone formation while promoting osteoclast differentiation and bone resorption; Kdm7a knockout increases bone mass and prevents OVX-induced bone loss (97). This finding identifies a single epigenetic enzyme that coordinately modulates both arms of bone remodeling. However, whether KDM7A exhibits consistent substrate specificity across osteoblasts, osteoclasts, and osteocytes remains unclear; selective KDM7A inhibitors have not yet been reported, and its pharmacological targetability awaits validation.

#### 4.2.2 Histone acetylation modification

Histone acetylation exerts bidirectional effects on bone metabolism through opposing enzymatic activities. At the level of acetylation-mediated promotion of osteogenesis, TGF-β1 acetylates Runx2 via p300, enhancing MMP13 transcription; p300 knockdown reduces Runx2 acetylation and MMP13 expression (98). Histone acetyltransferases (HATs) broadly regulate chromatin structure and the osteoblast-osteoclast balance, with non-coding RNAs also participating in this regulation (99). At the level of deacetylation-mediated suppression, prenatal caffeine exposure recruits histone deacetylase 11 (HDAC11) to the 11β-HSD2 promoter via the glucocorticoid receptor, inhibiting BMSC osteogenesis and establishing transgenerational bone programming (100).

Collectively, these findings establish a framework of “acetylation promotes bone, deacetylation suppresses bone.” However, the cell type-specific functions of HDAC11 in bone remain uncharacterized; how non-coding RNAs precisely regulate HAT/HDAC activity in osteoporosis requires further exploration.

#### 4.2.3 Synergistic conversion of acetylation and arginine methylation

A more complex regulatory mechanism involves the coordinated switching of distinct histone marks. The active vitamin D metabolite 1,25(OH)_2_D_3_ recruits the vitamin D receptor (VDR)/SRC-1 complex to the CYP24A1 promoter, triggering a switch that reduces the repressive H4R3me2s mark while increasing H3/H4 acetylation and H3R17me2a/H4R3me2a—thereby creating a transcriptionally active chromatin environment and upregulating CYP24A1 to maintain vitamin D homeostasis (101). This “acetyl-methyl coordinated switch” exemplifies how different histone modifications act synergistically rather than in isolation. However, whether this mechanism broadly regulates other bone metabolism genes and their pathological alterations in osteoporosis remains to be determined.

From a clinical translation perspective, histone-modifying enzymes have been successfully targeted in oncology—the HDAC inhibitor vorinostat (SAHA) is FDA-approved for cutaneous T-cell lymphoma, and several HDAC inhibitors (e.g., romidepsin, panobinostat) are in clinical use for hematologic malignancies. However, their application in osteoporosis remains largely unexplored. Key translational barriers include (1): target specificity—histone-modifying enzymes act genome-wide, and bone-selective delivery is undeveloped (2); safety—systemic HDAC inhibition causes thrombocytopenia, gastrointestinal toxicity, and fatigue in cancer patients, raising tolerability concerns for chronic osteoporosis treatment; (3) biomarkers—no validated markers exist to monitor bone-specific target engagement or histone modification status; and (4) compound availability—selective KDM7A inhibitors have not yet been reported.

Future directions should focus on: (1) bone-targeted HDAC or KDM7A inhibitors with improved selectivity; (2) exploring low-dose or intermittent HDAC inhibitor regimens for bone-specific benefits without systemic toxicity; (3) identifying circulating histone modification patterns as epigenetic biomarkers; and (4) investigating crosstalk between histone modifications, DNA methylation, and m6A RNA methylation for multi-target epigenetic strategies (**Table 2**).

**Table 2:** Histone modifications.

Category	Key regulatory factor	Main Mechanisms and effects
Histone methylation modification	Demethylase KDM7A	KDM7A demethylates Fap/Rankl, upregulating FAP/RANKL to impair bone formation; Kdm7a KO prevents OVX bone loss (97).
	p300(HAT)	TGF-β1/p300 acetylates Runx2 to drive MMP13 transcription (98).
Histone acetylation modification	HATs	Acetylation balances osteogenesis/osteoclastogenesis via chromatin; ncRNAs mediate this (99).
	HDAC11	Prenatal caffeine recruits HDAC11 to 11β-HSD2 via GR, impairing BMSC osteogenesis and programming intergenerational bone metabolism (100).
Synergistic Conversion of Acetylation and Arginine Methylation	1,25(OH)_2_D_3_	VDR/SRC-1 switches CYP24A1 from H4R3me2s to acetylation/arginine methylation, upregulating it to maintain vitamin D homeostasis (101).

### 4.3 RNA methylation

N6-methyladenosine (m6A) modification is the most abundant internal chemical modification in eukaryotic mRNA, regulated at the post-transcriptional level through the coordinated actions of “writers” (METTL3/14, WTAP), “erasers” (FTO, ALKBH5), and “readers” (YTHDF1/2, etc.), which collectively control mRNA stability, splicing, and translation efficiency. The critical role of m6A modification in bone metabolism regulation has become increasingly evident, with its dysregulation contributing to osteoporosis pathogenesis through four interconnected mechanisms: (1) m6A methyltransferases bidirectionally regulate osteogenic differentiation by modulating the mRNA stability and translation efficiency of key osteogenic genes (e.g., Grp78, RUNX2, TCF1, SMAD1); (2) m6A modification influences osteoclast differentiation, fusion, bone resorption, and migration by regulating the mRNA stability of Nos2, Atp6v0d2, Traf6, and CTSK; (3) the m6A demethylase ALKBH5 affects DNA damage repair and osteogenic differentiation through RAD51 mRNA stability regulation; and (4) non-coding RNAs (e.g., piR-36741) fine-tune osteogenic gene expression through interactions with m6A modification enzymes. These four layers represent the regulatory logic of m6A modification in “osteogenic differentiation regulation,” “osteoclast function regulation,” “demethylation dynamics,” and “non-coding RNA-m6A crosstalk,” collectively constituting a multi-level regulatory network governing bone metabolism.

#### 4.3.1 Regulation of osteoblast function by m6A methyltransferases (METTL3/14, WTAP)

The function of METTL3 in osteoblast differentiation is highly context-dependent, exerting opposing effects under different conditions. Under inflammatory stress, lipopolysaccharide (LPS) stimulation downregulates METTL3, and its knockdown stabilizes Grp78 mRNA via a YTHDF2-independent mechanism, activating endoplasmic reticulum (ER) stress pathways (PERK/eIF2α/ATF4/CHOP, IRE1α) and promoting osteoblast apoptosis while inhibiting proliferation and differentiation; the ER stress inhibitor 4-PBA reverses these effects (102). In osteoporotic BMSCs, reduced m6A and METTL3 correlate with impaired osteogenesis, and METTL3 overexpression restores osteogenic capacity through Wnt activation (103). In the context of miRNA regulation, METTL3 enhances RUNX2 and pre-miR-320 methylation while downregulating mature miR-320, promoting bone formation (104). As a negative regulator, METTL3 can also enhance MYD88 expression via m6A, activating NF-κB and inhibiting osteogenesis; its deletion relieves this inhibition (105). Under pathological metabolic conditions, high glucose and high lipid upregulate METTL3, enhancing ASK1 m6A modification and activating ASK1-p38 signaling to induce osteoblast ferroptosis (106).

METTL14 promotes osteogenesis through two identified mechanisms: it upregulates TCF1 via m6A modification, promoting RUNX2 and bone mass (107); it also modifies SMAD1 mRNA, which is recognized by IGF2BP1 to enhance SMAD1 stability and promote BMSC osteogenesis (108). WTAP, another m6A writer, promotes pri-miR-181a/c maturation via YTHDC1 recognition, leading to SFRP1 suppression and Wnt activation, thereby enhancing BMSC osteogenesis while inhibiting adipogenesis (109).

Collectively, these findings reveal that the same methyltransferase (METTL3) can exert opposite functions depending on cellular context. However, the mechanisms governing this functional switching remain poorly understood; most findings are derived from *in vitro* models, with human bone validation insufficient.

#### 4.3.2 Regulation of osteoclast function by m6A methyltransferases (METTL3/14)

METTL3 also plays context-dependent roles in osteoclasts. Under inflammatory conditions, METTL3 is downregulated; its knockdown enhances Nos2 mRNA stability via YTHDF1, promoting iNOS/NO production and inducing mitochondrial dysfunction and apoptosis, thereby inhibiting osteoclast differentiation (110). In contrast, during osteoclast differentiation, METTL3 is upregulated; its knockdown enhances Atp6v0d2 stability via YTHDF2, promoting osteoclast fusion while inhibiting resorption, and impedes Traf6 nuclear export, reducing TRAF6 protein and downstream MAPK, NF-κB, and PI3K-AKT signaling (111). Follicle-stimulating hormone (FSH) upregulates METTL3 through CREB phosphorylation, enhancing CTSK m6A methylation and promoting osteoclast migration (112). METTL14 suppresses osteoclastogenesis through m6A methylation of NFATc1, with exosomal delivery protecting bone mass (113).

This functional duality—METTL3 loss suppressing osteoclast differentiation under inflammation versus its presence being essential for normal fusion and signaling during differentiation—suggests that targeting METTL3 requires careful strategy design.

#### 4.3.3 Regulatory role of m6A demethylases (FTO, ALKBH5)

ALKBH5-mediated RAD51 mRNA demethylation reduces its stability, exacerbating DNA damage and inhibiting osteoblast differentiation; ALKBH5 knockdown alleviates OVX-induced bone loss (114). This study reveals the critical role of m6A dynamics in maintaining genomic stability and osteogenesis. However, the cell type-specific functions of ALKBH5 in bone and its synergistic relationships with other m6A regulators remain unclear.

#### 4.3.4 Interaction and regulatory mechanisms between non-coding RNA and m6A

The piR-36741-PIWIL4 complex interacts with METTL3, preventing m6A modification of BMP2 mRNA, thereby promoting BMP2 expression and osteogenesis; piR-36741 administration alleviates OVX-induced osteoporosis (115). This study is the first to link piRNA with m6A regulation in bone metabolism. However, piR-36741 expression changes in osteoporosis patients and its biomarker potential require large-scale clinical validation.

From a clinical translation perspective, no drugs targeting m6A machinery have been approved for any indication. However, first-wave small-molecule inhibitors have entered preclinical development: the METTL3 inhibitor STM2457 has shown efficacy in acute myeloid leukemia models, and FTO inhibitors (e.g., Dac51, CS1) have demonstrated anti-tumor activity. Their application in bone diseases remains unexplored. Key barriers include (1): target specificity—m6A regulators act globally, with unpredictable multi-tissue effects; (2) drug delivery—achieving sufficient bioavailability in bone tissue, especially the marrow microenvironment, is challenging; (3) safety—long-term systemic m6A modulation may interfere with normal brain and immune functions; and (4) biomarkers—no validated m6A-based markers exist for monitoring bone-specific target engagement.

Future directions should prioritize: (1) bone-targeted m6A modulators or exosome-based delivery systems; (2) exploring intermittent or localized m6A modulation for bone-specific benefits without systemic toxicity; (3) identifying circulating m6A signatures as biomarkers for patient stratification; and (4) investigating crosstalk between m6A, DNA methylation, and histone modifications for multi-target epigenetic strategies (**Tables 3**, **4**).

**Table 3:** RNA methylation.

Category	Key regulatory factor	Main mechanisms and effects
Regulation of Osteogenic Function by m6A	METTL3	METTL3 regulates osteogenesis via ER stress, Wnt, MYD88/NF-κB, and p38/ferroptosis (102, 104–106).
	METTL14	METTL14 promotes osteogenesisvia m6A-TCF1/RUNX2 and IGF2BP1-stabilized SMAD1 (107, 108).
	WTAP	WTAP/YTHDC1 activates Wnt via miR-181a/c-SFRP1 to promote BMSC osteogenesis (109).
Regulation of Osteoclast Function by m6A Methyltransferase	METTL3	METTL3 loss stabilizes Nos2 (inflammation) and Atp6v0d2 (differentiation); FSH upregulates METTL3 to stabilize CTSK and promote migration (110–112).
	METTL14	METTL14 methylates NFATc1 to inhibit osteoclasts; exosome delivery saves bone (113).
m6A demethylase	ALKBH5	ALKBH5 demethylates RAD51, impairing osteogenesis; its knockdown alleviates OVX bone loss (114).
Non-coding RNA and m6A interaction	piR-36741	piR-36741 blocks BMP2 m6A via PIWIL/METTL3 to promote osteogenesis; its delivery alleviates OVX osteoporosis (115).

**Table 4:** Molecular mechanisms and translational potential in osteoporosis: a comprehensive summary.

Mechanism	Key molecules	Involved cell type	Experimental model	Human evidence	Therapeutic target	Translational maturity
RANKL/RANK/OPG Axis	RANKL, RANK, OPG, Denosumab (22–24)	Osteoclasts, osteoblasts	OVX mice, in vitro BMMs	Clinical trials (Denosumab approved)	RANKL-neutralizing antibody (Denosumab)	Approved
Wnt/β-catenin Signaling	β-catenin, GSK-3β, LRP5/6, SOST, Romosozumab (39–52)	Osteoblasts, MSCs, osteocytes	Multiple transgenic mouse models, OVX	Clinical trials (Romosozumab approved)	Anti-SOST antibody (Romosozumab), DKK1 inhibitors	Approved
Piezo1/2 Mechanosensing	Piezo1/2, YAP/TAZ, NFATc1 (78–84)	Osteocytes, osteoblasts, MSCs, macrophages	Conditional knockout mice, fluid shear stress models	None	Piezo1 agonists (e.g., Yoda1 derivatives)	Preclinical
Metabolic Reprogramming (Osteoblasts)	ME2, HIF-1α, O-GlcNAc, PDK1, Glut1, Pfkfb3 (70–73)	Osteoblasts, MSCs	Osteoblast cell lines, diabetic mouse models	None	Glycolysis activators (e.g., Pfkfb3 overexpression)	Early-stage exploratory
Metabolic Reprogramming (Osteoclasts)	PRMT6, Glut1, glycolytic enzymes (74–77)	Osteoclasts	OVX mice, RAW264.7 cells	None	PRMT6 inhibitors, glycolysis inhibitors	Preclinical
DNA Methylation	DNMT1/3a/3b, TET1/2/3, Nrf2, ZEB1/2 (90–96)	MSCs, osteoblasts	Aged mice, OVX mice, progeroid models	None	DNMT inhibitors (e.g., SGI-1027), exercise intervention	Preclinical
Histone Modifications	KDM7A, p300/HATs, HDAC11, SAHA (97–105)	Osteoblasts, osteoclasts	OVX mice, in vitro osteogenic differentiation	Approved in oncology (SAHA)	KDM7A inhibitors, HDAC inhibitors	Preclinical (in bone)
RNA Methylation (m6A)	METTL3/14, WTAP, ALKBH5, YTHDF1/2 (106–115)	Osteoblasts, osteoclasts, MSCs	OVX mice, LPS-induced inflammation models	None	METTL3 inhibitors (e.g., STM2457)	Preclinical
Natural Products & Multi-Target Compounds	Curcumin, piperlongumine, kurarinone, agrimophol, icariin, TFRD, etc. (25–30, 49–57)	Osteoclasts, osteoblasts, MSCs	OVX mice, in vitro cell models	None	Lead compounds (under medicinal chemistry optimization)	Preclinical / Lead discovery
Osteocyte Senescence & SASP	SOST, RANKL, FGF23, DKK1, IL-6, SASP factors (116–120)	Osteocytes, MSCs	Aged mice, OVX models	None	Senolytics / Senomorphics	Preclinical
Mitochondrial Quality Control	Sirt3, PINK1/Parkin, FtMt (121, 122)	Osteoblasts, MSCs	AGEs-induced senescence models	None	Mitochondrial protectants	Early-stage exploratory
Calcium Signaling & Mitochondria	MCU, TRPV6, IGF-1/PI3K/AKT (34, 35, 120–123)	Osteoclasts, osteoblasts	OVX mice, in vitro cell models	None	MCU inhibitors (e.g., ruthenium red derivatives)	Preclinical

## 5 Organelle interactions and osteocyte aging

Osteocytes, stellate cells derived from osteoblasts, sense mechanical forces, regulate mineral metabolism, and coordinate osteoblasts/osteoclasts to maintain bone homeostasis. Their dysregulation leads to phosphate disorders or impaired bone coupling, making them therapeutic targets in osteoporosis. Key mechanisms include: Osteocytes are the most abundant cell type in bone tissue, constituting 90–95% of all adult skeletal cells (116). They are derived from osteoblasts that become embedded within the bone matrix during the mineralization process (117). These stellate cells form an extensive communication network through their dendritic processes, enabling them not only to sense mechanical loading, coordinate osteoblastic and osteoclastic activities, and regulate mineral metabolism, but also to exert systemic regulatory effects by secreting multiple key signaling molecules, including sclerostin (SOST), RANKL, fibroblast growth factor 23 (FGF23), and DKK1 (118–120). Within the dynamic equilibrium of bone remodeling, osteocytes precisely regulate local bone formation by modulating the intensity of SOST-mediated inhibition of the Wnt signaling pathway, while simultaneously influencing osteoclast differentiation and activation through dynamic adjustment of the RANKL/OPG ratio. This dual regulatory function establishes osteocytes as central integrators of bone metabolic homeostasis. Osteocyte dysfunction can lead to phosphate metabolic disorders, uncoupling of bone formation and resorption, and reduced bone quality. Notably, the discovery of SOST/sclerostin as an endogenous antagonist of the Wnt pathway directly drove the development and clinical application of the anti-sclerostin monoclonal antibody Romosozumab—the first osteoporosis therapeutic with dual actions of promoting bone formation and inhibiting bone resorption—thereby conceptually validating the feasibility of targeting osteocyte-derived signaling molecules as therapeutic targets.

### 5.1 Calcium homeostasis and mitochondrial function regulation

The active vitamin D analog ED-71 attenuates osteoblast senescence by improving ER-mitochondrial calcium homeostasis: it upregulates SERCA2 expression, reduces the p-PERK/PERK ratio to alleviate ER stress, and activates SIRT1-AMPK signaling, thereby improving mitochondrial function, promoting ATP production, and reducing the RANKL/OPG ratio in OVX rats (121). This study reveals a mechanistic link between vitamin D signaling, organellar calcium homeostasis, and osteoblast senescence. However, the long-term efficacy and safety of ED-71 in human osteoporosis require validation in large-scale trials.

### 5.2 Mitophagy and organelle interactions

Mitochondrial quality control is central to osteocyte function. Sirt3 clears damaged mitochondria through PINK1/Parkin-mediated mitophagy, thereby inhibiting MSC senescence and osteogenic dysfunction (122). Conversely, mitochondrial ferritin (FtMt) deficiency leads to osteoblast death via an excessive autophagy–ferroptosis axis, revealing pathological coupling between mitochondrial dysfunction and ferroptosis (123). These findings suggest that organelle crosstalk—particularly mitochondria-ER calcium homeostasis and mitochondria-lysosome autophagy pathways—plays critical roles in osteocyte aging. However, the precise molecular mechanisms of these networks in osteocytes and their differential roles across skeletal diseases remain poorly understood.

### 5.3 Integrated regulation of cellular senescence and SASP

Cellular senescence and its associated secretory phenotype (SASP) disrupt MSC function, impair the bone-cartilage microenvironment, and weaken regenerative capacity, thereby driving osteoporosis and intervertebral disc degeneration. Senolytic or senomorphic agents, combined with mitochondrial-autophagy restoration, represent an emerging strategy for reversing skeletal aging. Importantly, osteocyte senescence itself constitutes a major SASP source: senescent osteocytes secrete interleukin-6 (IL-6), MCP-1, and MMPs, which impair adjacent osteoblasts and enhance osteoclastogenesis via RANKL upregulation, ultimately establishing a positive feedback loop that begins with osteocyte senescence, propagates SASP-mediated microenvironmental deterioration, and culminates in enhanced bone resorption and further bone loss.

### 5.4 Clinical translation and future directions

The clinical success of Romosozumab provides a compelling paradigm for osteocyte-directed therapy. This humanized monoclonal antibody neutralizes sclerostin, relieving Wnt pathway suppression and exerting dual effects—stimulating bone formation while inhibiting resorption. Phase III trials demonstrated rapid BMD gains and significant fracture risk reductions versus placebo and alendronate. However, Romosozumab carries a boxed warning for cardiovascular events (myocardial infarction, stroke), restricting its use to patients without recent cardiovascular disease. This safety concern highlights the systemic effects of osteocyte-derived signals and the need for careful patient selection.

Key translational lessons from Romosozumab include (1): the need for predictive biomarkers to identify optimal candidates with the lowest cardiovascular risk; <<(>>2<<)>> the importance of therapeutic sequencing—whether Romosozumab should be followed by antiresorptives to maintain BMD gains; and (3) the potential for combination strategies targeting multiple osteocyte-derived signals (SOST, RANKL, DKK1, FGF23). Emerging osteocyte-directed therapies—including sclerostin vaccines and DKK1-neutralizing antibodies—are in preclinical or early clinical development [J]. Future research should also explore whether targeting osteocyte senescence can reduce SASP-mediated bone loss, potentially complementing or replacing SOST inhibition in age-related osteoporosis. However, current senolytic/senomorphic research in bone remains preclinical, and the lack of osteocyte-specific senescence markers limits precision targeting.

## 6 Future prospects

Based on the molecular mechanisms summarized in this review, future research in osteoporosis should advance along four strategic directions to achieve a shift from “symptom management” to “etiological intervention.” Each direction must be pursued with concurrent attention to its specific translational barriers and clear entry points for precision medicine.

### 6.1 Precise classification and biomarker development

Path-directed actions: A multi-omics-integrated molecular subtyping system should be established. Specifically, m6A-seq, single-cell sequencing, and other technologies should be employed for patient stratification, and combined diagnostic panels based on circulating miRNAs, SASP factors, or m6A modification profiles should be developed. Translational barriers include the high cost and lack of standardization in multi-omics data; most biomarker studies remain cross-sectional, lacking prospective cohort validation; and the scarcity of bone tissue-specific markers limits the ability of circulating biomarkers to accurately reflect local skeletal changes. Precision medicine opportunities: Large-scale longitudinal cohorts should be established, integrating multi-modal data through machine learning to systematically evaluate the clinical utility of candidate biomarkers in fracture risk prediction and therapeutic response monitoring.

### 6.2 Drug development targeting novel mechanisms

Three most promising intervention strategies: First, senolytics (e.g., dasatinib + quercetin) require clinical validation in senile osteoporosis, though challenges remain in bone tissue targeting, long-term safety, and drug resistance. Second, epigenetic modulators (targeting METTL3, KDM7A, etc.) can precisely regulate osteogenic or osteoclastic differentiation, but genome-wide off-target effects and the lack of mature bone cell-specific delivery strategies pose major safety and feasibility concerns. Third, mechanosensitive channel agonists (e.g., Yoda1 derivatives) could mimic mechanical stimulation to promote bone formation, yet current agonists lack subtype selectivity and have poor bioavailability, placing them at a considerable distance from clinical application.

### 6.3 Smart delivery and regenerative medicine

Path-directed actions: Bone-targeted nanocarriers should be developed for the precise delivery of siRNA or epigenetic drugs; smart biomaterial scaffolds responsive to the local microenvironment should be designed for controlled release of growth factors, or for loading mechanically preconditioned MSC-derived exosomes for bone defect repair. Core barriers include: suboptimal bone-targeting efficiency and poor *in vivo* stability of nanocarriers; insufficient systematic evaluation of long-term degradation behavior and biocompatibility of biomaterial scaffolds; and unresolved challenges in large-scale standardized exosome production and quality control. Without overcoming these bottlenecks, regenerative strategies will struggle to transition from bench to bedside.

### 6.4 Artificial intelligence and systems integration

Path-directed actions: artificial intelligence (AI) should be used to integrate clinical imaging, biomarkers, and multi-omics data to construct individualized fracture risk prediction models; machine learning should be employed to screen drug libraries for novel multi-target compounds capable of synergistically regulating the RANKL-Wnt-epigenetic network. Major challenges include: heterogeneity and annotation inconsistencies across multi-modal data modalities, which severely compromise model transferability; the “black box” nature of AI models, which limits their interpretability and acceptability in clinical decision-making; and the predominance of predictions without functional validation, creating a “more prediction, less validation” dilemma. Future efforts should establish standardized data-sharing platforms and strengthen iterative closed-loop cycles between AI prediction and experimental validation.

## 7.Conclusion

Future prevention and treatment strategies should shift from “symptom management” to “etiological intervention.” Through interdisciplinary collaboration, mechanistic insights should be translated into individualized diagnostic tools, novel therapeutic agents, and regenerative strategies, ultimately achieving the goal of restoring skeletal health.

## Glossary

4-PBA: 4-phenylbutyric acid
AI: artificial intelligence
AKT: protein kinase B
ALKBH5: AlkB homolog 5
ALP: alkaline phosphatase
AMPK: AMP-activated protein kinase
ATF4: activating transcription factor 4
BMD: bone mineral density
BMP: bone morphogenetic protein
BMSC: bone marrow mesenchymal stem cell
CB2: cannabinoid receptor type 2
CHOP: C/EBP homologous protein
CREB: cAMP response element-binding protein
CTSK: cathepsin K
DKK1: Dickkopf-related protein 1
DNMT: DNA methyltransferase
eIF2α: eukaryotic translation initiation factor 2 alpha
ER: endoplasmic reticulum
ERK: extracellular signal-regulated kinase
FAK: focal adhesion kinase
FGF23: fibroblast growth factor 23
FSH: follicle-stimulating hormone
FTO: fat mass and obesity-associated protein
Glut1: glucose transporter 1
GPX4: glutathione peroxidase 4
GSK-3β: glycogen synthase kinase-3 beta
HAT: histone acetyltransferase
HDAC: histone deacetylase
HIF-1α: hypoxia-inducible factor 1-alpha
HO-1: heme oxygenase-1
IGF-1: insulin-like growth factor 1
IL-6: interleukin-6
iNOS: inducible nitric oxide synthase
IRE1α: inositol-requiring enzyme 1 alpha
JNK: c-Jun N-terminal kinase
KDM7A: lysine demethylase 7A
KEAP1: Kelch-like ECH-associated protein 1
lncRNA: long non-coding RNA
LPS: lipopolysaccharide
LRP5/6: low-density lipoprotein receptor-related protein 5/6
m6A: N6-methyladenosine
MAPK: mitogen-activated protein kinase
MCP-1: monocyte chemoattractant protein-1
MCU: mitochondrial calcium uniporter
METTL: methyltransferase-like
miRNA: microRNA
MMP: matrix metalloproteinase
MSC: mesenchymal stem cell
NFATc1: nuclear factor of activated T cells c1
NF-κB: nuclear factor kappa-B
NO: nitric oxide
NOX: NADPH oxidase
NRF2: nuclear factor erythroid 2-related factor 2
OCN: osteocalcin
OP: osteoporosis
OPG: osteoprotegerin
OVX: ovariectomy/ovariectomized
PERK: protein kinase RNA-like endoplasmic reticulum kinase
PI3K: phosphoinositide 3-kinase
piRNA: Piwi-interacting RNA
PRMT: protein arginine methyltransferase
RANKL: receptor activator of nuclear factor-κB ligand
ROS: reactive oxygen species
RUNX2: runt-related transcription factor 2
SAHA: suberoylanilide hydroxamic acid
SASP: senescence-associated secretory phenotype
SIRT: sirtuin
SOST: sclerostin
TCF: T-cell factor
TET: ten-eleven translocation
TGF-β: transforming growth factor-beta
TRAF6: TNF receptor-associated factor 6
TRPV6: transient receptor potential vanilloid 6
VDR: vitamin D receptor
Wnt: Wingless-related integration site
WTAP: Wilms tumor 1-associating protein
YAP: Yes-associated protein
YTHDF: YTH domain-containing family protein
