# Correction: Molecular mechanisms and translational prospects in osteoporosis

**DOI:** 10.3389/fendo.2026.1954028

**Published:** 2026-08-20

**Authors:** Ping Xie, Jie Cai, Zhengjie Yu, Junjie Zhou, Jianping Wang

**Affiliations:** State Key Laboratory of New Targets Discovery and Drug Development for Major Diseases, Gannan Innovation and Translational Medicine Research Institute, Gannan Medical University, Ganzhou, China

**Keywords:** bone remodeling, molecular mechanisms, osteoporosis, research progress, therapeutic targets

Affiliations 1 and 2 were erroneously published as “1. State Key Laboratory of New Targets Discovery and Drug Development for Major Diseases, Ganzhou, China 2. Gannan Innovation and Translational Medicine Research Institute, Gannan Medical University, Ganzhou, China”. This affiliation has now been corrected to “State Key Laboratory of New Targets Discovery and Drug Development for Major Diseases, Gannan Innovation and Translational Medicine Research Institute, Gannan Medical University, Ganzhou, China”.

The original version of this article has been updated.

