# Supplementary material for: Molecular mechanisms and translational prospects in osteoporosis
Source: Front Endocrinol (Lausanne). 2026 Jul 22;17:1907271. doi: 10.3389/fendo.2026.1907271 (PMC13437411; doi:10.3389/fendo.2026.1907271)
Supplement: Supplementary file 1 [file Table1.docx]

**Supplementary Table 1: The search strategies of databases**

| Search Module | Search Query |
| --- | --- |
| #1 Osteoporosis | ("Osteoporosis"[Mesh] OR osteoporosis[Title/Abstract] OR "Bone Diseases, Metabolic"[Mesh] OR bone loss[Title/Abstract] OR low bone mass[Title/Abstract] OR osteopenia[Title/Abstract]) |
| #2 Bone Remodeling | ("Bone Remodeling"[Mesh] OR bone remodeling[Title/Abstract] OR bone resorption[Title/Abstract] OR bone formation[Title/Abstract] OR osteoclastogenesis[Title/Abstract] OR osteogenesis[Title/Abstract] OR "Osteoblasts"[Mesh] OR "Osteoclasts"[Mesh]) |
| #3 RANKL/RANK/OPG Axis | ("RANK Ligand"[Mesh] OR RANKL[Title/Abstract] OR "Receptor Activator of Nuclear Factor-kappa B"[Mesh] OR RANK[Title/Abstract] OR "Osteoprotegerin"[Mesh] OR OPG[Title/Abstract] OR Denosumab[Title/Abstract]) |
| #4 Wnt/β-catenin Signaling | ("Wnt Signaling Pathway"[Mesh] OR Wnt[Title/Abstract] OR beta-catenin[Title/Abstract] OR β-catenin[Title/Abstract] OR LRP5[Title/Abstract] OR LRP6[Title/Abstract] OR SOST[Title/Abstract] OR sclerostin[Title/Abstract] OR Romosozumab[Title/Abstract] OR GSK-3 beta[Title/Abstract]) |
| #5 Mechanosensing and Piezo | ("Mechanotransduction, Cellular"[Mesh] OR mechanotransduction[Title/Abstract] OR "Piezo1"[Supplementary Concept] OR Piezo1[Title/Abstract] OR Piezo2[Title/Abstract] OR YAP[Title/Abstract] OR TAZ[Title/Abstract] OR "YAP-Signaling Proteins"[Mesh] OR fluid shear stress[Title/Abstract]) |
| #6 Energy Metabolism Reprogramming | ("Energy Metabolism"[Mesh] OR energy metabolism[Title/Abstract] OR metabolic reprogramming[Title/Abstract] OR glycolysis[Title/Abstract] OR oxidative phosphorylation[Title/Abstract] OR "HIF-1 alpha"[Mesh] OR HIF-1α[Title/Abstract] OR "PRMT6"[Supplementary Concept] OR PRMT6[Title/Abstract] OR "Glucose Transporter Type 1"[Mesh] OR Glut1[Title/Abstract]) |
| #7 Epigenetic Regulation | ("Epigenesis, Genetic"[Mesh] OR epigenetic[Title/Abstract] OR "DNA Methylation"[Mesh] OR DNA methylation[Title/Abstract] OR "Histone Code"[Mesh] OR histone modification[Title/Abstract] OR "RNA Methylation"[Mesh] OR RNA methylation[Title/Abstract] OR m6A[Title/Abstract]) |
| #8 Osteocyte Senescence | ("Osteocytes"[Mesh] OR osteocyte[Title/Abstract] OR "Cellular Senescence"[Mesh] OR cellular senescence[Title/Abstract] OR SASP[Title/Abstract] OR "Senotherapeutics"[Mesh] OR senolytics[Title/Abstract] or senomorphics[Title/Abstract]) |
| #9 Mitochondria & Calcium | ("Mitochondria"[Mesh] OR mitochondria[Title/Abstract] OR "Calcium Signaling"[Mesh] OR calcium signaling[Title/Abstract] OR mitophagy[Title/Abstract] OR "Uncoupling Agents"[Mesh] OR "Ferroptosis"[Mesh] OR ferroptosis[Title/Abstract]) |
| #10 Natural Products | ("Biological Products"[Mesh] OR natural product[Title/Abstract] OR phytochemical[Title/Abstract] OR plant extract*[Title/Abstract] OR traditional Chinese medicine[Title/Abstract] OR (curcumin[Title/Abstract]) OR (icariin[Title/Abstract])) |
| Limits Applied | Filters: English or Chinese language; Humans; Animals; Clinical Trial; Review; Systematic Review; from database inception to 2026. |
